# Evaluation of the Relationship Between *BDNF* Val66Met Gene Polymorphism and Attention Deficit Hyperactivity Disorder: A Meta-Analysis

**DOI:** 10.3389/fpsyt.2022.888774

**Published:** 2022-04-28

**Authors:** Shufang Mei, Wencai Chen, Sijing Chen, Yani Hu, Xiaoyan Dai, Xiujun Liu

**Affiliations:** Department of Psychiatry, Wuhan Mental Health Center, Wuhan Hospital for Psychotherapy, Wuhan, China

**Keywords:** BDNF, Val66Met, polymorphism, attention deficit hyperactivity disorder, meta-analysis, risk

## Abstract

**Background:**

Attention deficit hyperactivity disorder (ADHD) is a common neurobehavioral disorder in childhood. Brain-derived neurotrophic factor (BDNF) is widely distributed in the central nervous system and plays an important role in neural development. Despite several previous studies have examined the association between the Val66Met polymorphism BDNF and ADHD, the results are conflicting.

**Objective:**

This study aimed to evaluate the association between Val66Met polymorphism and ADHD in case-control and transmission disequilibrium test (TDT) studies using a meta-analysis.

**Methods:**

Keywords “rs6265” or “Val66Met” and “Attention deficit hyperactivity disorder” were used to search in the PubMed, Embase, Web of Science, Wanfang, and China National Knowledge Infrastructure databases before April 2021. Genotype data were extracted to calculate odds ratios (ORs) and 95% confidence intervals (CIs).

**Results:**

Fifteen studies, comprising of 8,692 samples (containing 4,364 cases, 4,328 controls) and 1,578 families were included and results demonstrated that rs6265 was not associated with susceptibility to ADHD (OR = 0.95, 95% CI: 0.87–1.04, *P* = 0.291). Stratified analyses by study design, ethnicity, and sample size further supported that rs6265 was not associated with ADHD.

**Conclusion:**

The present study shows that the polymorphism of the *BDNF* Val66Met gene is not associated with susceptibility to ADHD.

## Introduction

Attention deficit hyperactivity disorder (ADHD) is a common neurobehavioral disorder in childhood (1), characterized by inattention, impulsivity, and hyperactivity, and usually accompanied by cognitive impairment, conduct disorder, and other mental disorders. According to recent meta-analysis including 175 eligible worldwide studies, the prevalence of ADHD is approximately 7.2% (2). Recent research has indicted that ADHD is not limited to childhood, but can also occur during adolescence and adulthood. For adolescents, ADHD symptoms often include internet addiction, increased smoking rates, drug abuse, alcohol-related problems, and high-risk sexual behaviors (3), whereas adult patients often face conduct-related problems and criminality (4). Given diverse and continuous negative effects on patients and the serious economic burden on families and society (5), ADHD is a major public health problem. Despite the etiology of ADHD is not fully understood, it is thought to be caused by both genetic and environmental factors. Decades of research on case–control, twin, and family studies have identified the genetic factors influencing the development of ADHD, and reported the mean heritability of ADHD to be up to 74% (6).

The brain-derived neurotrophic factor *(BDNF*) gene maps to human chromosome 11p14.1 (7). It contains four exons (I–IV) associated with distinct promoters located in the 5’ terminus, and only one (exon V) located in the 3’ terminus, which encodes the mature BDNF protein (8). Both mRNA and protein of *BDNF* are widely distributed in the central nervous system, particularly in the hippocampus and cerebral cortex (9). BDNF is a key protein that regulates neuronal survival and growth, and plays an important role in neural development and the maintenance of normal brain function (10). Several polymorphisms located in *BDNF* have been identified to be associated with ADHD, of which the polymorphism rs6265 is the most widely studied. Rs6265, also called Val66Met or G196A, leads to a Val to Met substitution at position 66 in the BDNF pro-domain that disrupts its transport and secretion, resulting in reduced cell surface expression (11, 12). Despite numerous studies that have examined the relationship between rs6265 and ADHD risk, the results are controversial and inconclusive. Whereas some studies have found that the Val allele is significantly associated with ADHD (13–16), other studies could not replicate these positive results (17–27), controversial results may be due to population stratification, false-positive results, and insufficient statistical power.

To overcome limitations of inadequate sample sizes, improve statistical power, and reduce false-positive results, we performed a meta-analysis to integrate results from both case-control (CC) and transmission disequilibrium test (TDT) studies for the association of rs6265 in *BDNF* with ADHD risk.

## Methods

### Literature Search

We performed a comprehensive search in the PubMed, Embase, Web of Science, Wanfang, and China National Knowledge Infrastructure databases to identify all potentially relevant studies using the keywords “rs6265,” “Val66Met,” and “attention deficit hyperactivity disorder.” The literature search was updated on April 30, 2021, and articles in English and Chinese were included for further analysis.

### Inclusion Criteria

The inclusion criteria for the studies were as follows: (1) published in English or Chinese; (2) CC or TDT studies; (3) evaluation of the association between rs6265 in *BDNF* and ADHD risk, (4) clear definition of ADHD, and (5) availability of data necessary for calculating odds ratios (ORs) with 95% confidence intervals (CIs). Reviews, case reports, case-only studies, animal studies, simple commentaries, and studies without sufficient data were excluded. When more than one study had overlapping data, only the study with the largest sample size was selected.

### Data Extraction

All data were extracted from the original studies by two independent investigators, and disagreements were resolved by discussion. The following information was extracted from the selected studies: authors, publication year, ethnicity and country of the study population, study design, diagnostic criteria for ADHD, sample size, counts of alleles in case and control groups in CC studies, and number of transmitted alleles from heterozygous parents to affected offspring in TDT studies.

### Statistical Analysis

The association between rs6265 and the risk of ADHD was evaluated using integrated ORs and 95% CIs. The between-study heterogeneity was measured using a Q-statistic test (28). When the *P*-value of the Q statistic test was smaller than 0.10, indicating significant heterogeneity across studies, a random-effects model was used (29). Otherwise, fixed effects model was applied (30). Stratified analysis was carried out according to study design (CC study and TDT study), ethnicity (Asian and European populations), and sample size (large sample-size subgroup: number of cases in CC studies or number of families in family based studies > 200; small sample-size: subgroup: number of cases in CC study or number of families in family based study < 200). Sensitivity analysis was performed by recalculating the ORs after removing each study to evaluate the stability of the results. Publication bias was investigated using a funnel plot and Egger’s test using the “catmap” and “metaphor” package in R software (31). A *P*-value of < 0.05 was considered statistically significant.

## Results

### Characteristics of Included Studies

**FIGURE 1:**
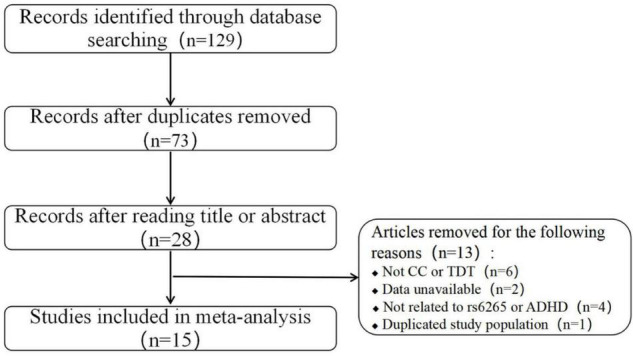
Flow diagram of the study selection used in this meta-analysis.

The literature search and study selection procedures are presented in a flow chart in Figure 1. A total of 129 records were identified through the above search strategy, and 28 records remained after removing the duplications and scanning the titles and abstracts. Of the remaining 28 records, 13 were further excluded according to the selection criteria: not CC or TDT studies (six studies), lack of sufficient data (two studies), not related to rs6265 or ADHD (four studies), and data duplication (one study). In addition, two records (15, 23) applied both CC and TDT designs in overlapping subjects. In these two instances, only CC studies were included in the overall meta-analysis because of the larger population, but the data from TDT designs were still used for the stratified analysis. After applying these inclusion criteria, 15 studies (seven conducted in Asian populations, seven in European populations, and one in a mixed population) containing 4,364 cases, 4,328 controls, and 1,578 families were retained for the meta-analysis (13–19, 21–23, 25–27). The detailed characteristics and allele information of the included studies are shown in Tables 1, 2.

**TABLE 1:** Main characteristics of studies included in the meta-analysis.

References	Country	Ethnicity	Study design	Case/Control (family)	Diagnostic criteria
					
Luo et al. (17)	Chinese	Asian	CC	195/263	DSM-V
Kwon et al. (18)	Korean	Asian	CC	178/158	DSM-IV
Liu et al. (19)	Chinese	Asian	CC	184/161	DSM-IV
Tzang et al. (20)	Chinese	Asian	TDT	285	DSM-IV
Li et al. (15)	Chinese	Asian	CC, TDT	1809/951 (1247)	DSM-IV
Cao et al. (16)	Chinese	Asian	CC	114/96	DSM-IV
Cho et al. (21)	Korean	Asian	CC	202/159	DSM-IV
Aureli et al. (13)	Italy	Caucasian	CC	37/80	DSM-IV
Sánchez-Mora et al. (22)	Germany	Caucasian	CC	607/840	DSM-IV
Sánchez-Mora et al. (22)	Netherlands	Caucasian	CC	191/486	DSM-IV
Sánchez-Mora et al. (22)	Norway	Caucasian	CC	436/496	DSM-IV
Sánchez-Mora et al. (22)	Spain	Caucasian	CC	211/425	DSM-IV
Lanktree et al. (23)	Canadian	Caucasian	CC, TDT	117/117 (80)	DSM-IV
Xu et al. (24)	United Kingdom	Caucasian	TDT	180	DSM-IV
Xu et al. (24)	Chinese	Asian	TDT	212	DSM-IV
Schimmelmann et al. (25)	Germany	Caucasian	TDT	294	DSM-IV
Lee et al. (26)	Canadian	Caucasian	TDT	266	DSM-IV
Friedel et al. (27)	Germany	Caucasian	CC	83/96	DSM-IV
Kent et al. (14)	United Kingdom	Caucasian	TDT	341	DSM-IV

**TABLE 2:** Allele information from studies included in the meta-analysis.

Study	CC		TDT	HWE
	Case(^1^R/^2^NR)	Control(^1^R/^2^NR)	Transmitted ^1^R/Non-transmitted ^1^R	
Luo et al. (17)	200/190	259/267	–	YES
Kwon et al. (18)	168/188	144/172	–	YES
Liu et al. (19)	172/196	159/163	–	YES
Tzang et al. (20)	–	–	148/140	YES
Li et al. (15)	1698/1920	884/1018	887/360	YES
Cao et al. (16)	61/167	79/113	–	YES
Cho et al. (21)	185/219	152/166	–	YES
Aureli et al. (13)	10/64	56/104	–	YES
Sánchez-Mora et al. (22)	239/975	362/1318	–	YES
Sánchez-Mora et al. (22)	77/305	176/796	–	YES
Sánchez-Mora et al. (22)	176/696	189/803	–	YES
Sánchez-Mora et al. (22)	99/323	177/673	–	YES
Lanktree et al. (23)	31/203	43/191	18/33	YES
Xu et al. (24)	–	–	41/45	–
Xu et al. (24)	–	–	60/63	–
Schimmelmann (25)	–	–	144/138	YES
Lee et al. (26)	–	–	79/73	YES
Friedel et al. (27)	30/136	35/157	–	YES
Kent et al. (14)	–	–	36/58	YES

*HWE: Hardy–Weinberg equilibrium; TDT, transmission disequilibrium test.*

*^1^R:represents risk allele.*

*^2^NR:stands for non-risk allele.*

### Combined Results of Case-Control Transmission Disequilibrium Test Studies

**FIGURE 2:**
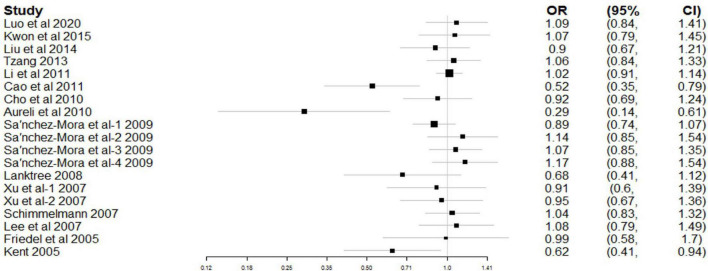
Forest plot for the association between rs6265 and attention deficit hyperactivity disorder (ADHD) risk.

Figure 2 shows the combined results of CC and TDT studies for the study of rs6265 association with ADHD risk. A random-effects model was applied because of significant heterogeneity (χ^2^ = 33.19, *P*_*heterogeneity*_ = 0.016). In the overall meta-analysis, no significant association was observed between rs6265 and ADHD (OR = 0.95, 95% CI: 0.87–1.04, *P* = 0.291).

### Stratified Analysis

As shown in Table 3, a stratified analysis was first performed by grouping the included studies by study design. However, both the CC and TDT subgroups were significantly heterogeneous (CC: χ^2^ = 27.34, *P*_*heterogeneity*_ = 0.007; TDT: χ^2^ = 125.97, *P*_*heterogeneity*_ < 0.001). No significant association was observed between rs6265 and ADHD in either subgroup (CC: OR = 0.94, 95% CI: 0.84–1.06, *P* = 0.309; TDT: OR = 1.00, 95% CI: 0.67–1.52, *P* = 0.981).

**TABLE 3:** Meta-analysis for associations between *BDNF* Val66Met and ADHD.

Stratification factor	Number of studies	Case/Control (family)	OR (95% CI)	*P*	Heterogeneity		*P*-value for Egger’s test
					Q statistic	*P*_heterogeneity_	
Overall	19	4364/4328 (1578)	0.95 (0.87–1.04)	0.291	33.19	0.016	0.041
Study design							
CC	13	4364/4328	0.94 (0.84–1.06)	0.309	27.34	0.007	0.120
TDT	8	2905	1.00 (0.67–1.52)	0.981	125.97	< 0.001	<0.001
Ethnicity							
Asian	8	2682/1788 (497)	0.99 (0.91–1.07)	0.765	11.23	0.129	0.236
Caucasian	11	1682/2423 (1081)	0.93 (0.81–1.08)	0.361	21.83	0.016	0.041
Sample size							
Large	10	3265/2871 (1398)	1.00 (0.93–1.07)	0.993	9.11	0.427	0.448
Small	9	1099/1457 (180)	0.85 (0.69–1.05)	0.132	22.56	0.004	0.001

Then, the data were stratified according to ethnicity. No heterogeneity was found the Asian population (χ^2^ = 11.23, *P*_*heterogeneity*_ = 0.129), but still existed in the Caucasian population (χ^2^ = 21.83, *P*_*heterogeneity*_ = 0.016). Ethnicity-specific meta-analysis indicated no association between rs6265 and ADHD in either the Asian (OR = 0.99, 95% CI: 0.91–1.07, *P* = 0.765) or Caucasian (OR = 0.93, 95% CI: 0.81–1.08, *P* = 0.361) populations.

Finally, the data were stratified according to sample size. The large sample-size subgroup showed no heterogeneity (χ^2^ = 9.11, *P*_*heterogeneity*_ = 0.427), however the small-sample-size subgroup still demonstrated significant heterogeneity (χ^2^ = 22.56, *P*_*heterogeneity*_ = 0.004). No significant association was observed between rs6265 and ADHD in either subgroup (large sample-size: OR = 1.00, 95% CI: 0.93–1.07, *P* = 0.993; small sample size: OR = 0.85, 95% CI: 0.69–1.05, *P* = 0.132).

### Sensitivity Publication Bias Analyses

**FIGURE 3:**
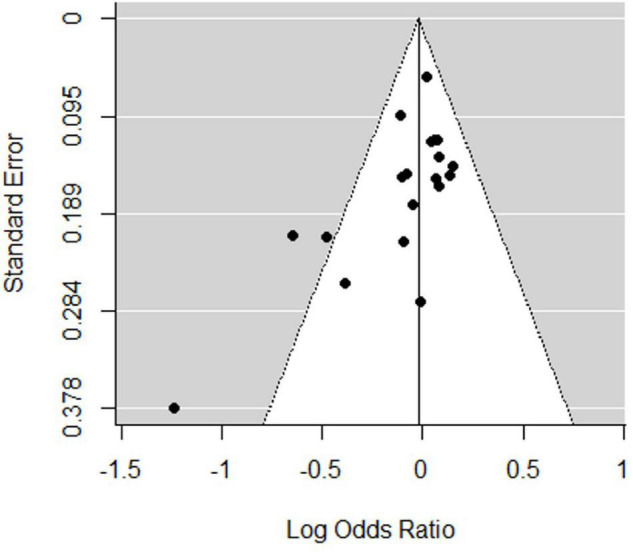
Funnel plot of the association between rs6265 and attention deficit hyperactivity disorder (ADHD) risk.

Sensitivity analysis was performed by systematically removing a study from the analysis to assess the influence of each study on the pooled OR. This demonstrated relatively robust results for rs6265, with no reverse outcomes. As reflected by the funnel plot (Figure 3) and Egger’s test, significant publication bias was detected for rs6265 (*P* = 0.041), which may be related to the fact that negative results are not commonly published.

## Discussion

A previous meta-analysis reported a negative association between the rs6265 polymorphism of the *BDNF* gene and ADHD in European and Asian populations (22, 32). However, subsequent studies failed to replicate this result (13, 16–20). This meta-analysis aimed to investigate this controversy, including CC and TDT studies. The results of this meta-analysis indicated null associations between *BDNF* rs6265 polymorphism and ADHD risk in all populations. Further analyses revealed that *BDNF* rs6265 polymorphism was not associated with CC and TDT study design, Asian and European populations, and large and small sample-size populations.

As the most widely distributed and most important neurotrophic factor in the brain (33), the role of BDNF in brain neurodevelopment has received widespread attention. BDNF has been reported to regulate the plasticity of neurons and play a protective role in the regeneration and repair of neurons, through binding to tyrosine kinases B (TrkB), and mediating three signal transduction pathways, including phospholipase, mitogen-activated protein kinase/extracellular signal-regulated protein kinase, and phosphoinositol 3 kinase (34–36). Although the underlying mechanism of ADHD is unclear, the most studied models of pathophysiology of ADHD involved frontal-striatal-cerebellar circuits and ventral striatal-limbic circuits (37). Dopamine is an important neurotransmitter in the central nervous system, and its content in brain is one of the pathogenesis of ADHD. Inhibition of AC/cAMP/PKA pathway leads to feedback reduction of DA, resulting in cognitive impairment (38). Dopamine transporter is essential to maintaining Dopamine balance, Dopamine transporter knockout rats altered the BDNF system in the dorsolateral striatum (39). In addition, studies using patients serum, animal models, and genetic studies have suggested that *BDNF* is closely related to ADHD (40–42). BDNF serum concentration has been reported to correlate with the clinical symptoms of neurodevelopmental disorders such as reduced intelligence, behavioral problems, and intellectual impairment in preschoolers (43). A recent study uncovered that the level of serum BDNF in ADHD children was significantly higher than that in healthy controls, and that it had a positive relation with the severity of symptoms (33). However, another study reported the opposite result among adults with ADHD (44). In line with this result, *BDNF* knockout mice showed aggressive behavior similar to that of ADHD compared with wild littermates (45). Although these studies indicate that BDNF plays a role in the development of ADHD, the underlying mechanism remains unclear.

A common exonic Val66Met substitution within the pro-region of *BDNF* has been widely implicated in ADHD by affecting intracellular transport and activity-dependent BDNF secretion (12, 46). Val66Met was detected in the hippocampus and prefrontal cortex, which are the regions with the most abundant expression of BDNF (47), the central regions of neural plasticity and adaptation related to learning and memory (48), as well as important brain regions in the development of ADHD (12). Therefore, due to the role of BDNF Val66Met in these processes, its relationship with ADHD has attracted increasing attention, but the results are not completely consistent. The results of this meta-analysis do not support the role of the Val66Met polymorphism in susceptibility to ADHD, which was not consistent with the results of epidemiological and functional studies (23, 49–51). TDT studies, such as those conducted by Xu et al. (24) and Tzang et al. (20) did not observe significant associations in the Taiwanese population. Similarly, other studies have reported negative results in the Caucasian population (25, 26). In contrast, Kent et al. (14) and Lanktree et al. (23) reported excessive transmission of allele G of *BDNF* with a strong paternal effect. In line with this, Li et al. (15) found excessive transmission of allele G in female Chinese ADHD patients. Finally, the CC studies proceeded by Friedel et al. (27), Cho et al. (21), and Luo et al. (17) were unable to demonstrate the effect of Val66Met on susceptibility to ADHD, but Aureli et al. (13) reported a positive result.

This study aimed to explore the relationship between *BDNF* Val66Met polymorphism and ADHD, including eight CC studies, five TDT studies, and two mixed trials. Our results suggest that the *BDNF* rs6265 polymorphism may not be a genetic factor for ADHD. The reasons underlying discrepancies regarding the role of rs6265 in ADHD susceptibility are unclear. Stratified analysis revealed that the heterogeneities in the Caucasian group and in the small sample group were increased in comparison with the other groups, which might be related to the fact that negative results are not easy to publish. One potential explanation for discrepancies in the different association studies is the variable distribution of the assessed phenotype in each sample. In this study, we assessed the proportions of ADHD-C (44.4–100%), ADHD-I (7–50.4%), ADHD-HI (0–27%). In Chinese population, the proportion of ADHD-C, ADHD-I, and ADHD-HI were 44.4–79.49%, 15.38–50.4%, 4.8–5.31%, respectively; whereas they were 54.0–100%, 7–24.0%, 0–27.0%, respectively, in the European population. Clinical heterogeneity between samples may mask a real association. Although it is currently unclear how clinical heterogeneity may relate to these differences, additional studies on *BDNF* Val66Met polymorphism on the different subtypes of response to ADHD are still warranted. Another possible underlying explanation relies on the gender imbalance of the samples. The prevalence of ADHD in men is significantly higher than that in women; however, only one study in the Chinese Han population was evaluated the association considering the gender of patients. The role of gender in ADHD susceptibility needs to be investigated in future research.

Despite these limitations, this updated meta-analysis integrating CC and TDT studies helped to clarify the association between *BDNF* Val66Met polymorphism and susceptibility to ADHD and suggested that rs6265 may not contribute to the risk of ADHD.

## Data Availability Statement

The original contributions presented in the study are included in the article/supplementary material, further inquiries can be directed to the corresponding author.

## Author Contributions

SM and WC designed the study and did the literature review. SM, SC, YH, and XD did the statistical analysis and prepared the manuscript draft. XL and WC did quality control and contributed to the revisions in depth for the manuscript. All authors contributed to the article and approved the submitted version.

## Conflict of Interest

The authors declare that the research was conducted in the absence of any commercial or financial relationships that could be construed as a potential conflict of interest.

## Publisher’s Note

All claims expressed in this article are solely those of the authors and do not necessarily represent those of their affiliated organizations, or those of the publisher, the editors and the reviewers. Any product that may be evaluated in this article, or claim that may be made by its manufacturer, is not guaranteed or endorsed by the publisher.
